# Hepatitis-C Infection: Are we really committed to eliminate? Could it become the second Polio for Pakistan?

**DOI:** 10.12669/pjms.36.7.2804

**Published:** 2020

**Authors:** Lubna Kamani, Baseer Sultan Ahmad, Hamid Ali Kalwar

**Affiliations:** 1 Lubna Kamani, MBBS, FCPS, MRCP (UK), FRCP (London), FACG. Associate Professor and Director GI Residency Program, Department of Gastroenterology, Liaquat National Hospital, Karachi, Pakistan; 2 Baseer Sultan Ahmad. FCPS(Gastro). Assistant Professor, Department of Gastroenterology. Liaquat National Hospital, Karachi, Pakistan; 3 Hamid Ali Kalwar. FCPS(Gastro). Consultant, Murshid Hospital, Karachi, Pakistan

**Keywords:** Chronic liver disease, Hepatitis C, Hepatitis C elimination, Polio, Pakistan, Viral hepatitis

## Abstract

Pakistan’s hepatitis C virus (HCV) burden is one of the highest in the world. Around eight million people live with HCV in Pakistan according to a National Hepatitis Survey. Most HCV-infected people are unaware of their infection status culminating in delayed diagnosis and treatment, progressing to end stage liver disease, cirrhosis, and hepatocellular carcinoma (HCC), thereby raising the disease load for a developing country with limited resources. Blood transfusions and injections with reused syringes lead to increased HCV rates in Pakistan. According to a survey viral infections like hepatitis C, hepatitis B and HIV were not screened in more than half of the blood transfusions done in Pakistan. Hepatitis C elimination requires financial support from the local government and private organizations, commitment from civil societies across the world and a dedicated political will. Without defining effective planning and strategy it is our fear that it could become the second Polio for Pakistan.

First discovered in 1989, the hepatitis C virus (HCV) has become the most widespread global health epidemic, with recent estimates showing more than 185 million infected people worldwide.^1^ It is one of the leading cause of morbidity and mortality worldwide.

Persons infected with this infection are often asymptomatic, and approximately 50-80% are unaware of their illness. HCV can progress to end stage liver disease, cirrhosis and hepatocellular carcinoma (HCC) in one third of infected individuals over a period of 20–30 years, with an estimated half a million people dying yearly from these complications. Approximately eight million are infected in Pakistan,^2^ which is second largest burden across the globe after China.^3^

In developing countries like Pakistan, due to lack of insufficient implementation of standard guidelines on blood transfusion, reuse of syringes and needles for tattooing and ear piercing, and insufficient sterilization of potentially contaminated surgical and dental equipment are the key reasons for HCV transmission.^3^-^5^ A National survey done in 2007 found out that around 4.8 per cent of the population were exposed to HCV.^4^

HCV prevalence in another survey conducted in 2018 highlights the prevalence in adult population, blood donors, pregnant women, children, patients with different diseases, and intravenous drug users were 11.55%, 10.10%, 4.65%, 1.5%, 24.97%, 51.0% respectively. Genotype 3a is the most common genotype and found in 63% of infected cases in Pakistan.^6^

Blood transfusions are one of the major contributors for higher rate of HCV in Pakistan and approximately half of the blood donors are not screened for HCV, hepatitis B virus (HBV) and Human Immunodeficiency Virus (HIV).^5^ Majority of the blood donors are paid or people who inject drugs thus increase the risk for HCV exposure, mainly in private sector because of lack of oversight by local government and lax regulation. This fact reinforces the need for a reform of blood transfusion in Pakistan which, according to the World Health Organization, involves improving health regulation, uplifting infrastructure and promoting literacy through cultural norms.

Due to a lack of field research, unreliable and dearth of epidemiological data, underreporting, fragile health services, lack of education, awareness, and negligence are to some extent responsible for the HCV spread in Pakistan.

The introduction of interferon free all oral direct antiviral activity (DAA) treatment has achieved groundbreaking success. It is well tolerated with cure rate of over 90%. DAA has played a critical role in eradication strategy.^7^ Recently, a global health sector strategy (GHSS) has been implemented by the World Health Organization (WHO) to eliminate HCV. This policy commits countries to actively pursue the removal of viruses. The goals for HCV elimination include, 80% reduction in incident infections and a 65% reduction in mortality associated with HCV by 2030.^7^ Pakistan’s government introduced a National Strategic Framework on Hepatitis [NHSF] in October 2017. To respond to viral hepatitis, successful implementation of this depends on concerted Federal and Provincial efforts from all stakeholders in the health. Pakistan is the country with the largest number of per capita therapeutic injections per year. Research by Pasha et al showed that HCV cases were 11.9 times more prevalent in household members receiving more than 4 injections a year.^8^ In his report, Lim AG et al reported that, in Pakistan, without timely intervention and management, the HCV burden will have increased by two-thirds to 1.1 million new cases annually.^9^

According to the progress report of the WHO on access to hepatitis C care, in 2016, 161,000 HCV patients received treatment in Pakistan (predominantly from the private sector).^10^ A recent modeling study indicates that Pakistan needs to increase its number of HCV treatments (up to 880,000 treatments per year) in order to meet the GHSS goals for viral hepatitis. The number of interventions can be reduced to (to 525,000 a year), by targeting individuals like drug abusers, cirrhotic and through scaling up preventive measures.^9^ We need to have comprehensive National guidelines by the Government stake holders for prevention, mass screening, linkage to care thus ultimately leading to micro elimination of HCV.

HCV elimination is slowly gaining momentum in various parts of the world. Currently, nine countries in the world are on the right track to achieve HCV elimination by 2030. Unfortunately, with the current state of affairs this target seems un-achievable in Pakistan.^11^

There is a dire need to speed up the diagnosis of HCV and to identify the missing millions. Only about one in five people afflicted with HCV were diagnosed globally in 2016. Non-governmental organizations (NGOs) are playing a key role in the global fight against hepatitis, however the funding provided for the research of the NGOs on eliminating hepatitis in Pakistan seems to be largely inadequate.

Hepatitis C infection is a global challenge, in Pakistan polio eradication programs still haven’t achieved their targets, despite the campaign being started in 1974. Pakistan, Nigeria and Afghanistan are the three remaining countries where polio is still an endemic.^12^ Fragmented health care system with insufficient training and lack of awareness are the main hindrances behind un-successful polio campaign. Elimination of hepatitis infection requires prevention and seriousness at a grass root level, awareness should be created among the people so as they could apply these measures. With the availability of a new treatment there is chance of effective elimination. The only way to stop this menace of hepatitis C is to emphasize the mode of transmission, through public awareness programs, educating the public regarding modes of transmission and in addition, the government should become more proactive in providing education in schools, which can be effective partner in spread of awareness and success of HCV elimination drive.

## CONCLUSION

The control of hepatitis C epidemic requires combined effort by government and NGOs, together with political will, financial investment, well-structured long term planning and also support from civil societies around the globe. Without effective strategy we dread that HCV could become the second Polio for Pakistan.

### Author’s Contribution:

**LK:** Conceived the idea, design, critical review and approval of manuscript. She is also responsible and accountable for the accuracy or integrity of the study.

**BSA:** Literature search and writing first draft of manuscript.

**HAK:** Extended literature search, editing and finalizing second draft of manuscript.

## References

[1] Mohd Hanafiah K, Groeger J, Flaxman AD, Wiersma ST (2013). Global epidemiology of hepatitis C virus infection:new estimates of age-specific antibody to HCV seroprevalence. Hepatol.

[2] Bosan A, Qureshi H, Bile KM, Ahmad I, Hafiz R (2010). A review of hepatitis viral infections in Pakistan. J Pak Med Assoc.

[3] Khan AA, Khan A (2010). The HIV epidemic in Pakistan. J Pak Med Assoc.

[4] Chaudhry M, Rizvi F, Afzal M, Ashraf M, Niazi S, Beg A (2010). Frequency of risk factors for Hepatitis B (HBV) and Hepatitis C Virus (HCV). Ann Pak Inst Med Sci.

[5] Alaei K, Sarwar M, Alaei A (2018). The urgency to mitigate the spread of hepatitis C in Pakistan through blood transfusion reform. Int J Health Policy Manag.

[6] Zaheer HA, Waheed U (2014). Legislative reforms of the blood transfusion system in Pakistan. Transfus Med.

[7] World Health Organization. Global Health Sector Strategy on Viral Hepatitis 2016–2021 Towards ending viral hepatitis.

[8] Pasha O, Luby SP, Khan AJ, Shah SA, McCormick JB, Fischer-Hoch SP (1999). Household members of hepatitis C virus-infected people in Hafizabad, Pakistan:infection by injections from health care providers. Epidemiol Infect.

[9] Lim AG, Qureshi H, Mahmood H (2018). Curbing the hepatitis C virus epidemic in Pakistan:the impact of scaling up treatment and prevention for achieving elimination. Int J Epidemiol.

[10] World Health Organization progress report on access to hepatitis C treatment (2018). Focus on overcoming barriers in low and middle income countries.

[11] Polaris Observatory Center for Disease Analysis.

[12] Global Polio Eradication Initiative >Key countries“.

